# A revision of the subgenus Dudaica Strand of the genus *Drosophila* Fallén, with descriptions of six new species (Diptera, Drosophilidae)

**DOI:** 10.3897/zookeys.781.27354

**Published:** 2018-08-09

**Authors:** Takehiro K. Katoh, Guang Zhang, Masanori J. Toda, Awit Suwito, Jian-Jun Gao

**Affiliations:** 1 State Key Laboratory for Conservation and Utilization of Bioresources in Yunnan, Yunnan University, 2 Cuihubeilu, Kunming, Yunnan 650091, China; 2 Hokkaido University Museum, Hokkaido University, N10, W8, Kita-ku, Sapporo 060-0810, Japan; 3 Zoology Division (Museum Zoologicum Bogoriense), Research Center for Biology-LIPI, Cibinong, Bogor 16911, Indonesia; 4 Laboratory of Ecology and Evolutionary Biology, Yunnan University, 2 Cuihubeilu, Kunming, Yunnan 650091, China

**Keywords:** China, DNA barcoding, Southeast Asia, taxonomy

## Abstract

The subgenus Dudaica Strand of the genus *Drosophila* Fallén has been known to comprise only two species: Drosophila (Dudaica) senilis Duda, 1926 (recorded from Indonesia, Philippines, Vietnam, Bhutan, and India) and *D.malayana* (Takada, 1976) (recorded from Malaysia). In the present study, this subgenus is revised, with *D.malayana* redescribed and six new species discovered and described from China, Malaysia, and Indonesia: *gracilipalpis* Katoh & Gao, **sp. n.**, *puberula* Katoh & Gao, **sp. n.**, *albipalpis* Katoh, Toda & Gao, **sp. n.**, *qiongzhouensis* Katoh & Gao, **sp. n.**, *orthophallata* Katoh, Toda & Gao, **sp. n.**, and *dissimilis* Katoh & Gao, **sp. n.** Both morphological and molecular data (DNA barcodes) are used to distinguish the above species. A key to species of this subgenus is provided.

## Introduction

Duda (1926) established the monotypic subgenus Macropalpus Duda (type species: *Drosophilasenilis* Duda, 1926 from Sumatra) in the genus *Drosophila* Fallén, and defined it by the following diagnostic characters: (1) palpus distinctly large, long and broad, lacking prominent setae, (2) scutellum large, apically broadly rounded, and (3) costal break turned inwards onto thickened end of R_1_. Later, Strand (1943) proposed *Dudaica* as a replacement name of the subgenus Macropalpus, since the name *Macropalpus* had been preoccupied. In a revision of the genus *Zygothrica* Wiedemann, Grimaldi (1990a) transferred *Z.malayana* Takada, 1976 from *Zygothrica* into Drosophila (Dudaica), referring to Takada’s (1976) original description, and mentioned that an undetermined species of *Dudaica*, but distinct from *malayana*, was present in New Guinea. According to previous records, *D.senilis* is widely distributed in the Oriental region, from not only Sumatra (the type locality) but also the Philippines and Java (Wheeler 1981), India (Gupta and Sundaran 1990), Bhutan (De and Gupta 1996), and Vietnam (Sidorenko 1996).

The phylogenetic position of the subgenus Dudaica remains unresolved. Grimaldi (1990b) proposed a revised phylogenetic classification of the family Drosophilidae, based on a cladistic analysis for a set of 120 species (including *D.senilis*) representing genera and subgenera of the family. Grimaldi’s final consensus cladogram placed *D.senilis* most close to D. (Drosophila) monochaeta Sturtevant, 1927, and both formed a cluster with *Idiomyia* s. lat. However, he was “not confident of the homologies for the two features suggesting this relationship [reduction in number of interfrontal setulae (ap. 67) and a reduced, simple spermatheca (ap. 217)]”. Yassin (2013) revised the subgeneric classification of *Drosophila* in light of molecular and morphological data, and proposed diagnoses for the subgenera, including *Dudaica*. However, his proposal that *Dudaica* is closely related to the genera *Hirtodrosophila* Duda, *Paraliodrosophila* Duda and *Zygothrica* was elicited solely by a single morphological trait, i.e., the shape of the “gonopods”, but not by molecular data.

In this paper, we revise the subgenus Dudaica, and add six new species discovered from China, Malaysia, and Indonesia to this subgenus, by identifying them with the aid of DNA sequences of the 658-bp barcoding region of the mitochondrial COI (cytochrome *c* oxidase subunit I) gene. We also redescribe the known species *D.malayana*, based on specimens newly collected from Malaysia and Indonesia. Finally, a key to all the eight species of *Dudaica* is given.

## Materials and methods

All specimens employed in the present study were collected from China, Malaysia, and Indonesia (Table 1). They were mostly captured from herb layer in forest by net sweeping, and preserved immediately in either 70% or 100% ethanol for morphological observation and DNA sequencing, respectively.

**Table 1.** List of species and specimens examined in the present study.

Species	Sex	Voucher # ^a^	Collection site	Elevation (m)	Collection date	GenBank accession #
*malayana* (Takada, 1976)	♂	#03903	Poring, Sabah, Malaysia	600	20.iii.2008	MH410612
	♂	#03904	Ditto	600	13.iii.2008	MH410613
	♀	n/a	Gunung Poteng, West Kalimantan, Indonesia	220	4.xii.1996	n/a
*gracilipalpis* sp. n.	♂	#00033	Xishuangbanna Tropical Botanical Garden, Mengla, Xishuangbanna, Yunnan, China	650	19.iii.2006	MH410614
	♂	#03423	Ditto	650	27–28.ix.2011	MH410620
	♂	#06001	Ditto	650	28.ix.2011	MH410624
	♂	#**00484**	Wangtianshu, Mengla, Xishuangbanna, Yunnan, China	670	22–25.iv.2007	MH410615
	♂	#00491	Ditto	670	30.ix.2011	MH410617
	♂	#03364	Ditto	670	30.ix.2011	MH410619
	♂	#03424	Ditto	670	30.ix.2011	MH410621
	♂	#03425	Ditto	670	30.ix.2011	MH410622
	♀	#00485	Ditto	670	22–25.iv.2007	MH410616
	♀	#00492	Ditto	670	30.ix.2011	MH410618
	♂	#03902	Bogor, West Java, Indonesia	260	14–15.xi.2009	MH410623
*puberula* sp. n.	♂	#**03365**	Xishuangbanna Tropical Botanical Garden, Mengla, Xishuangbanna, Yunnan, China	650	27–28.ix.2011	MH410626
	♀	#03426	Ditto	650	19.iii.2006	MH410631
	♂	#00480	Wangtianshu, Mengla, Xishuangbanna, Yunnan, China	670	22–25.iv.2007	MH410625
	♂	#03366	Ditto	670	30.ix.2011	MH410627
	♂	#03367	Ditto	670	30.ix.2011	MH410628
	♂	#03368	Ditto	670	30.ix.2011	MH410629
	♂	#03369	Ditto	670	30.ix.2011	MH410630
*albipalpis* sp. n.	♂	#**03908**	Cikaniki, Gunung Halimun, West Java, Indonesia	1050	4.xi.2009	MH410632
*qiongzhouensis* sp. n.	♂	#03310	Jianfengling National Nature Reserve, Ledong, Hainan, China	750	17–20.iv.2008	MH410633
	♂	#03311	Ditto	750	17–20.iv.2008	MH410634
	♂	#03312	Ditto	750	17–20.iv.2008	MH410635
	♂	#03418	Ditto	750	17–20.iv.2008	MH410639
	♂	#03419	Ditto	750	17–20.iv.2008	MH410640
	♂	#**03420**	Ditto	750	17–20.iv.2008	MH410641
	♀	#03313	Ditto	750	17–20.iv.2008	MH410636
	♀	#03314	Ditto	750	17–20.iv.2008	MH410637
	♀	#03315	Ditto	750	17–20.iv.2008	MH410638
	♀	#03422	Ditto	750	17–20.iv.2008	MH410642
*orthophallata* sp. n.	♂	#**00177**	Ulu Senagang, Crocker Range, Sabah, Malaysia	540	18.x.1999	n/a
	♀	#03905	Park Headquarters, Mt. Kinabalu, Sabah, Malaysia	1700	11.iii.2008	MH410644
	♀	#03906	Ditto	1700	11.iii.2008	MH410645
*dissimilis* sp. n.	♂	#**00430**	Hesong, Xiding, Menghai, Xishuangbanna, Yunnan, China	1900	7.iv.2011	MH410646

^a^ Numbers in bold indicate holotypes of new species.

Specimens were first identified as of the subgenus Dudaica, based on their overall resemblance to the two known species of this subgenus, *D.senilis* and *D.malayana*, especially in body color pattern, shape of palpus, and structures of male/female terminalia. The holotype specimen of *D.malayana* was examined for the reference. As for *D.senilis*, we referred to Duda’s (1926) original description, Gupta and Sundaran’s (1990) redescription of terminalia, and Grimaldi’s (1990b) character states in his cladistic analysis. All specimens were then sorted into known or putatively new species in light of morphology. For this, external morphology was examined, numbers of morphometric characters were measured, and detailed structures in male/female terminalia, head and mouth parts were observed by the same methods as in Li et al. (2014).

The specimens were then subjected to DNA barcoding analysis (Hebert et al. 2003), with total DNA extracted from a right hind- or mid-leg, or small piece(s) of abdominal tissue picked from the abdominal dissection cut, using the TIANamp^®^ Genomic DNA Kit. DNA sequences of the 658-bp barcoding region of the mitochondrial COI gene were then amplified with the Folmer et al.’s (1994) primer pair, following the procedures as in Li et al. (2014). The PCR products were purified and sequenced with ABI3730 sequencer. The obtained DNA sequences were edited and aligned in the SeqMan module of the DNAStar package (DNAStar Inc. 1996) and MEGA7 (Kumar et al. 2016), respectively. A molecular phylogenetic tree was constructed by using Bayesian Inference (BI) method in MrBayes v3.2.6 (Ronquist et al. 2012), with the sequence data partitioned into two subsets by codon position, i.e., 1st+2nd codon positions and 3rd codon position. In BI, two independent runs of MCMC with four chains each (three heated and one cold) were conducted simultaneously for 5,000,000 generations, and trees were sampled every 100 generations. The analysis was stopped after verifying convergence statistics using Tracer v1.6 (Rambaut et al. 2014), and the first 20% of the tree samples were discarded as burn-in. Nucleotide substitution model was determined for each data set using jModelTest 2.1.10 (Guindon and Gascuel 2003, Darriba et al. 2012) using the Bayesian Information Criterion (BIC; Schwarz 1978). In addition, we employed the Automatic Barcode Gap Discovery (ABGD; Puillandre et al. 2012) and the General Mixed Yule-coalescent (GMYC; Pons et al. 2006) analyses for the molecular species delimitation. The ABGD analysis was run on the web-interface (http://wwwabi.snv.jussieu.fr/public/abgd/abgdweb.html) with the default settings [*P*_min_ = 0.001, *P*_max_ = 0.1, Steps = 10, *X* (a proxy for minimum gap width) = 1.5, Nb bins (for distance distribution) = 20]. All three distances applicable in the web-interface, JC69 (Jukes and Cantor 1969), K2P (Kimura 1980), and simple distances (i.e., p-distances) were used for the analyses. The GMYC was performed using the package “splits” (http://r-forge.r-project.org/projects/splits) in R, with the single-threshold strategy and default scaling parameters. An ultrametric tree for the GMYC was generated by BEAST v2.4.5 (Bouckaert et al. 2014) using the Yule prior and the HKY (Hasegawa et al. 1985) with a proportion of invariable sites (+I) model, with 5,000,000 MCMC generations. In addition, the intra- and inter specific p-distances for the species in *Dudaica* were calculated with MEGA7 and summarized.

For species illustration, the external morphology and detailed structures of male and female terminalia, and head and mouth parts were microphotographed using a Dino-Lite^®^ Microscope Eyepiece Camera (ANMO Electronics Corporation). We followed McAlpine (1981) for morphological terminology, and Zhang and Toda (1992) for definitions of measurements and indices. The examined specimens are deposited in the following institutions:

**KIZ** Kunming Natural History Museum of Zoology, Kunming Institute of Zoology, Chinese Academy of Sciences, Kunming, China

**KPSP** Kinabalu Park, Sabah Parks, Sabah, Malaysia

**ITBC** Institute for Tropical Biology and Conservation, Universiti Malaysia Sabah, Kota Kinabalu, Sabah, Malaysia

**MZB**Museum Zoologicum Bogoriense, Bogor, Indonesia

**SEHU** Systematic Entomology, The Hokkaido University Museum, Hokkaido University, Sapporo, Japan

## Results

A total of 34 COI sequences of 658-bp were determined in this study (Table 1). We failed to determine the COI sequence for the male specimen #00177 (of *D.orthophallata* sp. n. to be described here), probably due to poor quality of the total DNA extracted from this specimen, which was collected in 1999. Also, one female specimen of *D.malayana*, which was the oldest one examined here, was not used for DNA analysis. The HKY+I model was selected for both of the ‘1st+2nd codon positions’ and ‘3rd codon position’ partitions as the best nucleotide substitution model for BI analysis.

**Figure 1.**
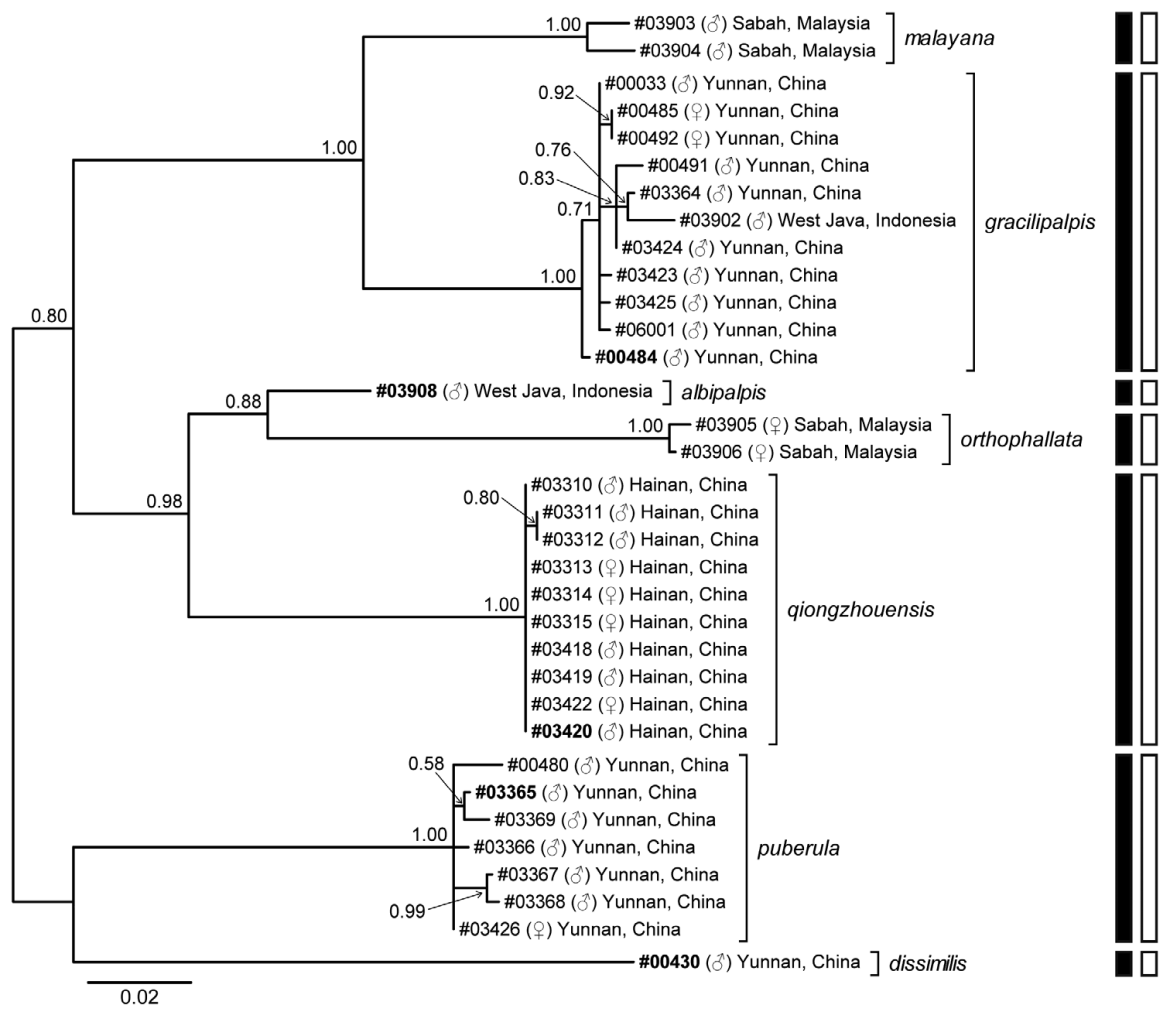
Bayesian tree of seven species of the subgenus Dudaica based on COI gene sequences, with indication of the results of molecular species delimitation by the ABGD (solid bars) and GMYC (open bars) analyses. Label of each operational taxonomic unit (OTU) is given in the format of “voucher number (gender) province of collection locality” (bold voucher numbers: holotype specimens). Numbers beside nodes are posterior probabilities.

Figure 1 illustrates the unrooted BI tree built with the 34 COI sequences and the results for the molecular species delimitation. The ABGD and GMYC analyses resulted in the same hypothesis: the studied sequences were sorted into seven hypothetical species, except for at *P* (prior intraspecific divergence) = 0.001 in ABGD using JC69 and K2P distances where eight species including a paraphyletic one were proposed. These seven Molecular Operational Taxonomic Units (hypothetical species) were supported by morphological data as well. In addition, the highest intraspecific (i.e., within-lineage) p-distance was 0.0163, while the lowest interspecific (i.e., among- lineage) p-distance was 0.0796, indicating a broad barcoding gap (Table 2). Thus, in consequence of integrative species delineation based on molecular and morphological data, we recognized seven (one known and six new) species within our studied samples of the subgenus Dudaica: *D.malayana* (Takada, 1976), *D.gracilipalpis* sp. n., *D.puberula* sp. n., *D.albipalpis* sp. n., *D.qiongzhouensis* sp. n., *D.orthophallata* sp. n., and *D.dissimilis* sp. n.

**Table 2.** Summary of intra- and interspecific p-distances.

Species	Intraspecific distance		Interspecific distance^b^						
	Mean (SE^a^)	Range	1	2	3	4	5	6	7
1. *malayana* (Takada)	0.0163 (n/a)	0.0163		0.0796−0.0906	0.1279−0.1378	0.1195−0.1248	0.1262−0.1356	0.1343−0.1392	0.1230−0.1244
2. *gracilipalpis* sp. n.	0.0057 (0.0014)	0.0000−0.0144	0.0832 (0.0106)		0.1260−0.1386	0.1125−0.1172	0.1225−0.1279	0.1431−0.1528	0.1293−0.1335
3. *puberula* sp. n.	0.0089 (0.0023)	0.0015−0.0152	0.1321 (0.0128)	0.1329 (0.0132)		0.0949−0.1004	0.1231−0.1262	0.1254−0.1336	0.1227−0.1277
4. *albipalpis* sp. n.	n/a	n/a	0.1221 (0.0129)	0.1143 (0.0129)	0.0964 (0.0114)		0.0844−0.0861	0.0870−0.0896	0.1215
5. *qiongzhouensis* sp. n.	0.0005 (0.0005)	0.0000−0.0015	0.1304 (0.0130)	0.1245 (0.0115)	0.1248 (0.0123)	0.0858 (0.0113)		0.1135−0.1184	0.1178
6. *orthophallata* sp. n.	0.0035 (n/a)	0.0035	0.1361 (0.0138)	0.1456 (0.0143)	0.1297 (0.0137)	0.0883 (0.0114)	0.1154 (0.0134)		0.1360−0.1385
7. *dissimilis* sp. n.	n/a	n/a	0.1237 (0.0133)	0.1313 (0.0127)	0.1260 (0.0126)	0.1215 (n/a)	0.1178 (0.0124)	0.1373 (0.0144)	

^a^ SE, standard error; ^b^ Values of mean p-distance (SE) below diagonal, value ranges of p-distance above diagonal.

Among them, *D.qiongzhouensis* sp. n. and *D.albipalpis* sp. n. are very similar in morphology to each other. The diagnoses for these species are supplemented with “pure” molecular diagnostic characters, which are defined as sites with fixed status in the COI sequence alignments within the focal species but differing from the other species (Sarkar et al. 2002, DeSalle et al. 2005) (Table 3).

**Table 3.** Selected diagnostic nucleotide sites (indicated in square brackets) for *D.albipalpis* sp. n. and *D.qiongzhouensis* sp. n. in the COI sequences. Sequences of the other five species in the subgenus Dudaica are shown for comparison.

Species	Sequence	Diagnostic nucleotide sites			
		92	226	391	589
*albipalpis* sp. n.	#03908	T	T	[C]	[T]
*qiongzhouensis* sp. n.	#03310	[C]	[C]	T	C
	#03311	[C]	[C]	T	C
	#03312	[C]	[C]	T	C
	#03313	[C]	[C]	T	C
	#03314	[C]	[C]	T	C
	#03315	[C]	[C]	T	C
	#03418	[C]	[C]	T	C
	#03419	[C]	[C]	T	C
	#03420	[C]	[C]	T	C
	#03422	[C]	[C]	T	C
*malayana* (Takada)	#03903	T	T	T	C
	#03904	T	T	T	C
*gracilipalpis* sp. n.	#00484	T	T	T	C
	#00033	T	T	T	C
	#00485	T	T	T	C
	#00491	T	T	T	C
	#00492	T	T	T	C
	#03364	T	T	T	C
	#03423	T	T	T	C
	#03424	T	T	T	C
	#03425	T	T	T	C
	#03902	T	T	T	C
	#06001	T	T	T	C
*puberula* sp. n.	#00480	T	T	T	C
	#03365	T	T	T	C
	#03366	T	T	T	C
	#03367	T	T	T	C
	#03368	T	T	T	C
	#03369	T	T	T	C
	#03426	T	T	T	C
*orthophallata* sp. n.	#03905	T	T	T	C
	#03906	T	T	T	C
*dissimilis* sp. n.	#00430	T	T	T	C

## Taxonomy

### Subgenus Dudaica

Taxon classificationAnimaliaDipteraLauxaniidae

Strand, 1943

Dudaica Strand, 1943: 212. New name for Macropalpus Duda.
Macropalpus Duda, 1926: 63. Type species: Drosophilasenilis Duda, 1926. Preoccupied by Macropalpus Ratzeburg, 1844 (Braconidae). Proposed as a subgenus.

#### Diagnosis.

**Figure 2.**
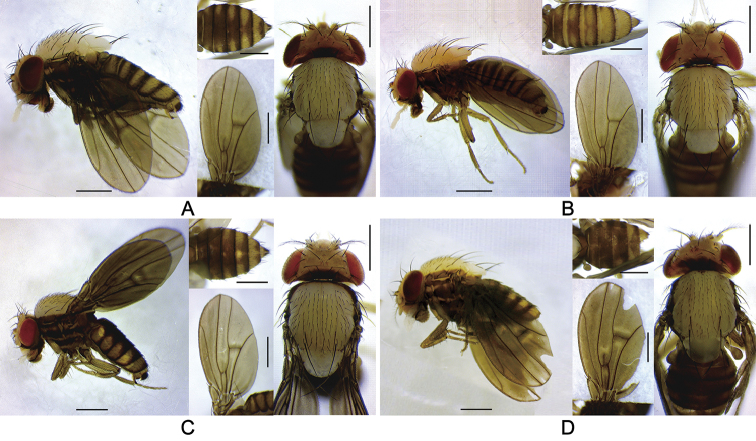
Left lateral habitus, thorax, wing, and abdomen of *Dudaica* species (part 1). **A***malayana* (Takada) (#03904) **B***gracilipalpis* sp. n. (paratype #03902) **C***puberula* sp. n. (holotype #03365) **D***albipalpis* (holotype #03908). Scale bars: 0.5 mm.

**Figure 3.**
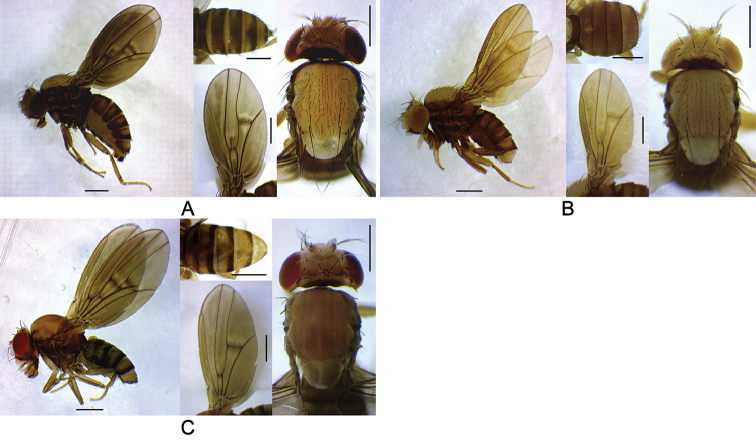
Left lateral habitus, thorax, wing, and abdomen of *Dudaica* species (part 2). **A***qiongzhouensis* (holotype #03420) **B***orthophallata* sp. n. (holotype #00177) **C***dissimilis* (holotype #00430). Scale bars: 0.5 mm.

**Figure 4.**
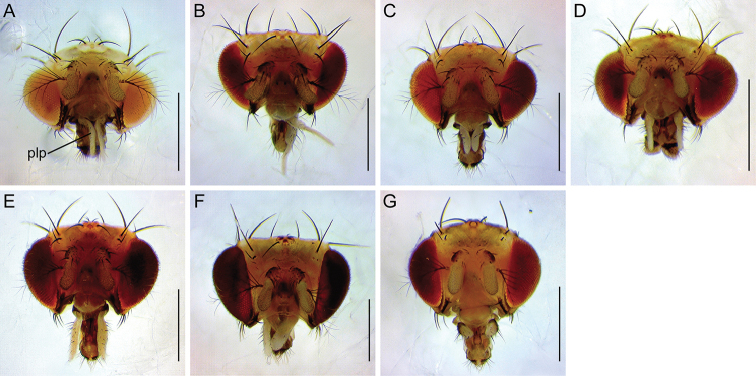
Head (anterior view). **A***malayana* (Takada) (#03903) **B***gracilipalpis* sp. n. (paratype #00492) **C***puberula* sp. n. (paratype #03366) **D***albipalpis* sp. n. (holotype #03908) **E***qiongzhouensis* sp. n. (paratype #03313) **F***orthophallata* sp. n. (paratype #03905) **G***dissimilis* sp. n. (holotype #00430). Abbreviation: plp = palpus. Scale bars: 0.5 mm.

Head, scutum, and scutellum mostly milky white, contrasting with mostly dark brown thoracic pleura (Figures 2–4). Scutellum large, more or less rounded apically in dorsal view (Figures 2, 3). Wing fuscous, somewhat wavy (Figures 2, 3).

#### Common characters.

**Figure 5.**
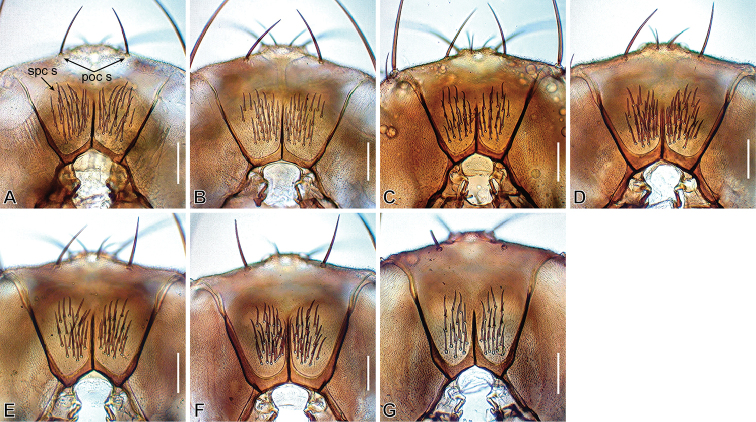
Postocciput (caudal view). **A***malayana* (Takada) (#03903) **B***gracilipalpis* sp. n. (paratype #00492) **C***puberula* sp. n. (paratype #03366) **D***albipalpis* sp. n. (holotype #03908) **E***qiongzhouensis* sp. n. (paratype #03313) **F***orthophallata* sp. n. (paratype #03905) **G***dissimilis* sp. n. (holotype #00430). Abbreviations: poc s = postocellar setae, spc s = supracervical setae. Scale bars: 0.1 mm.

**Figure 6.**
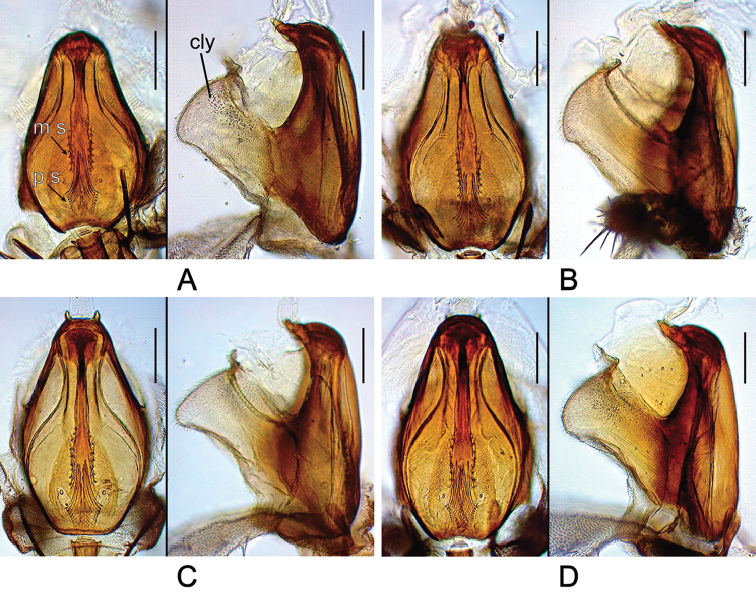
Cibarium of *Dudaica* species (part 1; dorsal and lateral views). **A***malayana* (Takada) (#03903) **B***gracilipalpis* sp. n. (paratype #00492) **C***puberula* sp. n. (paratype #03366) **D***albipalpis* sp. n. (holotype #03908). Abbreviations: cly = clypeus, m s = medial sensilla, p s = posterior sensilla. Scale bars: 0.1 mm.

**Figure 7.**
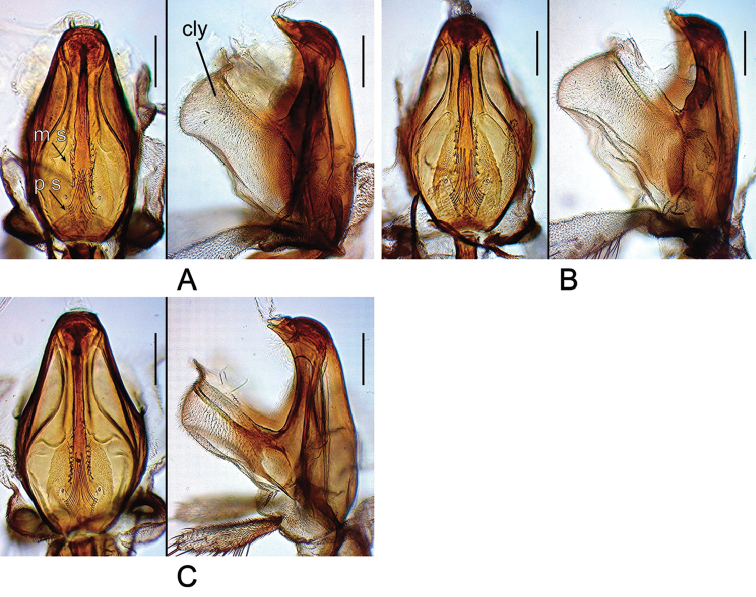
Cibarium of *Dudaica* species (part 2). **A***qiongzhouensis* sp. n. (paratype #03313) **B***orthophallata* sp. n. (paratype #03905) **C***dissimilis* (holotype #00430). Scale bars: 0.1 mm.

**Figure 8.**
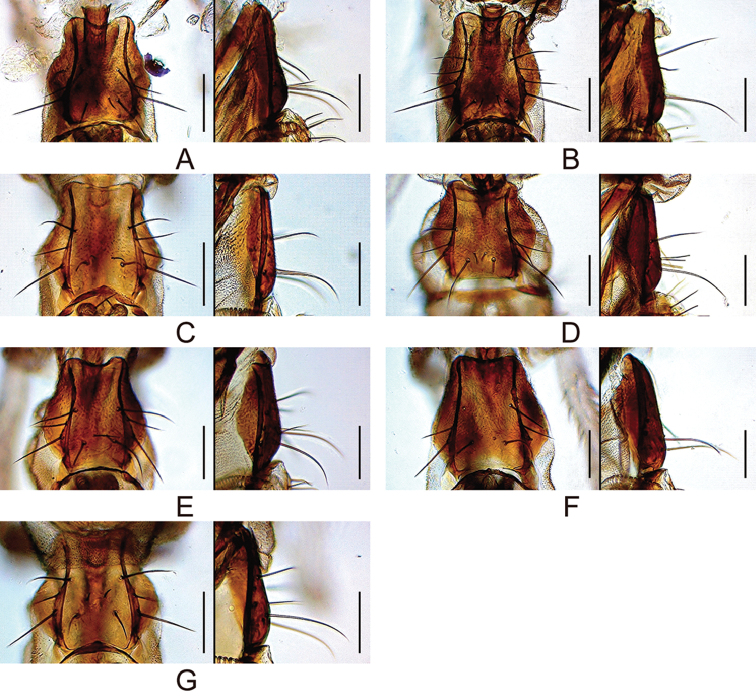
Prementum (ventral and lateral views). **A***malayana* (Takada) (#03903) **B***gracilipalpis* sp. n. (paratype #00492) **C***puberula* sp. n. (paratype #03366) **D***albipalpis* sp. n. (holotype #03908) **E***qiongzhouensis* sp. n. (paratype #03313) **F***orthophallata* sp. n. (paratype #03905) **G***dissimilis* sp. n. (holotype #00430). Scale bars: 0.1 mm.

**Figure 9.**
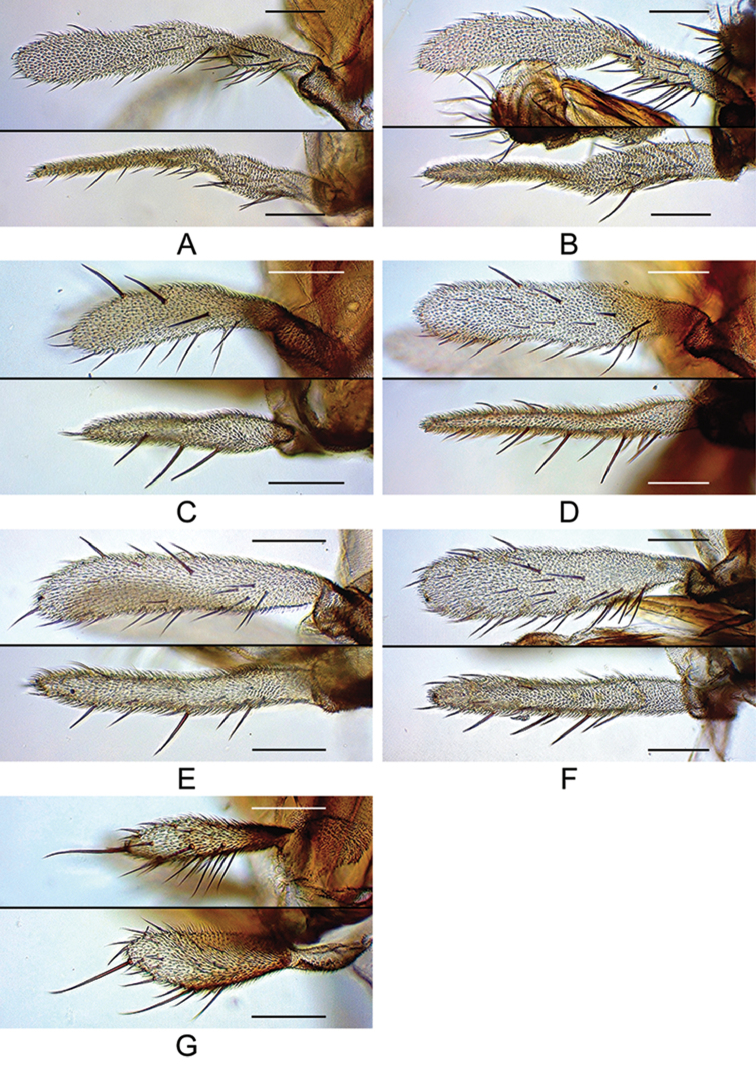
Palpus (lateral and dorsal views; only A is photographed at right side and flipped horizontally). **A***malayana* (Takada) (#03903) **B***gracilipalpis* sp. n. (paratype #00492) **C***puberula* sp. n. (paratype #03366) **D***albipalpis* sp. n. (holotype #03908) **E***qiongzhouensis* sp. n. (paratype #03313) **F***orthophallata* sp. n. (paratype #03905) **G***dissimilis* sp. n. (holotype #00430). Scale bars: 0.1 mm.

*Head* (Figures 2–9): Eye red, with dense interfacetal setulae; longest axis of eye slightly oblique (nearly rectangular in *dissimilis* sp. n.) against body axis. Ocellar triangle convex; ocellar setae inserted outside triangle made by ocelli. Anterior reclinate orbital seta situated slightly before proclinate orbital seta or just lateral to it (between proclinate and posterior reclinate orbital setae in *dissimilis* sp. n.) (Figure 4). Pedicel dorsolaterally dark brown (Figure 4); first flagellomere pubescent; arista with 6–8 dorsal and 2–4 ventral branches in addition to terminal bifurcation; terminal bifurcation moderate. Facial carina high, broad. Gena anteriorly dark brown. Occiput ventrally dark brown. Postgena medially dark brown. Postocellar setae present (Figure 5). Supracervical setae tapered, thin, apically curved and slightly blunt (Figure 5). Cibarium not thickened on anterior margin in lateral view (slightly thickened in *dissimilis* sp. n.); anterior portion slightly dilated; anterolateral corners slightly protruded; dorsal wall pear-shaped, with posterior portion oval; anterior sensilla four, arranged in square; medial sensilla apically sharp, arranged in anteriorly slightly convergent rows; posterior sensilla apically blunt, arranged in anteriorly divergent rows (Figures 6, 7). Clypeus thick at median portion (except for *dissimilis* sp. n.) (Figures 6, 7). Palpus distinctly large, long and broad (Figures 4, 9) (except for *dissimilis* sp. n.), pubescent, setigerous, distally flat (except for *dissimilis* sp. n.) (Figures 9). Prementum dark brown, swollen at the distal end in lateral view (Figure 8). Labellum with five pseudotracheae per side.

*Thorax* (Figures 2, 3): Postpronotal lobe with 1–3 prominent (lowermost the longest) and 0–4 short setae. Scutum narrowly dark brown along anteromost margin. Thoracic pleura mostly covered with broad, dark brown, more or less blurry, longitudinal stripes (except for *dissimilis* sp. n.). Basal scutellar setae divergent; apical scutellar setae cruciate. Acrostichal setulae in six, somewhat regular rows. Mid katepisternal seta much shorter than anterior and posterior ones, but distinct from setulae in row below it; caudoventral corner of katepisternum with one long, prominent seta.

*Wing* (Figures 2, 3) pale grayish yellow to grayish yellow, elliptic, rounded distally (not so elliptic in *dissimilis* sp. n.), wrinkled especially at basal portion of R_4+5_; longitudinal veins brown except for R_2+3_ (pale brown), basal section of M_1_ (dark brown), and CuA_1_ (dark brown) (except for *dissimilis* sp. n.); crossveins shaded at r-m and dm-cu; C_1_ setae two, subequal; R_2+3_ distally slightly curved to costa; R_4+5_ basally diverged from M_1_, distally nearly parallel with M_1_; M_1_ more or less sinuate; A_1_ well developed, as stout as other veins. Halter entirely grayish yellow to grayish brown.

**Figure 10.**
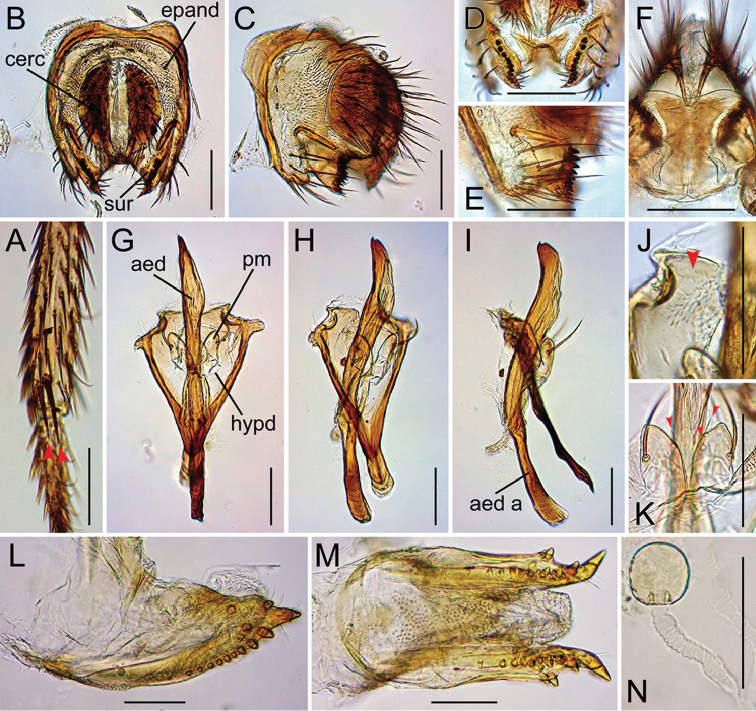
Drosophila (Dudaica) malayana (Takada) (**A, K** #03903 **B–J** #03904 **L–N** female specimen). **A** tibia of right foreleg (apical setae indicated with red arrowheads; ventral view) **B, C** periphallic organs (caudal and caudolateral view, respectively) **D** surstyli (caudoventral view) **E** surstylus and epandrial ventral lobe (caudolateral view) **F** tenth sternite and ventral protrusions of cerci (ventral view) **G–I** phallic organs (ventral, ventrolateral, and lateral view, respectively) **J** pubescence on caudolateral lobe of hypandrium (indicated with red arrowhead; caudoventral view) **K** sensilla (indicated with red arrowheads) and apical portion of parameres (ventral view) **L, M** oviscapt (lateral and ventral view, respectively) **N** spermatheca. Abbreviations: aed = aedeagus, aed a = aedeagal apodeme, cerc = cercus, epand = epandrium, hypd = hypandrium, pm = paramere, sur = surstylus. Scale bars: 0.1 mm.

**Figure 11.**
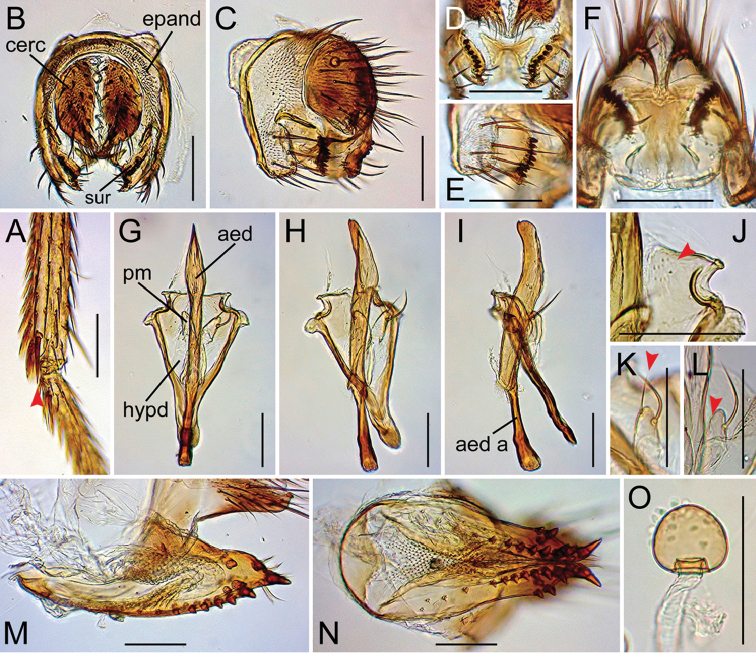
Drosophila (Dudaica) gracilipalpis sp. n. (**A, M–O** paratype #00492 **B–K** paratype #03902 **L** paratype #00491). **A** tibia of right foreleg (apical seta indicated with red arrowhead; ventral view) **B, C** periphallic organs (caudal and caudolateral view, respectively) **D** surstyli (caudoventral view) **E** surstylus and epandrial ventral lobe (caudolateral view) **F** tenth sternite and ventral protrusions of cerci (ventral view) **G–I** phallic organs (ventral, ventrolateral, and lateral view, respectively) **J** pubescence on caudolateral lobe of hypandrium (indicated with red arrowhead; caudoventral view) **K, L** sensilla (indicated with red arrowheads) and apical portion of parameres (ventral view; Indonesian and Chinese specimen, respectively) **M, N** oviscapt (lateral and ventral view, respectively) **O** spermatheca. Scale bars: 0.1 mm.

*Legs* (Figures 2, 3, 10A, 11A) pale grayish yellow to grayish brown. Preapical dorsal setae present on tibiae of all legs; apical setae present on fore- and mid-leg tibiae. Foreleg first tarsomere with one subproximal and one apical, short, black spines.

*Abdomen* (Figures 2, 3): Tergite 1 nearly entirely dark brown, 2 to 6 pale yellow, each with anterior and caudal dark brown bands; anterior bands medially sometimes interrupted; caudal bands medially and laterally extended anteriad (except for *dissimilis* sp. n.). Female tergite 7 nearly entirely pale yellow. Sternites somewhat quadrate, grayish brown to dark brown.

**Figure 12.**
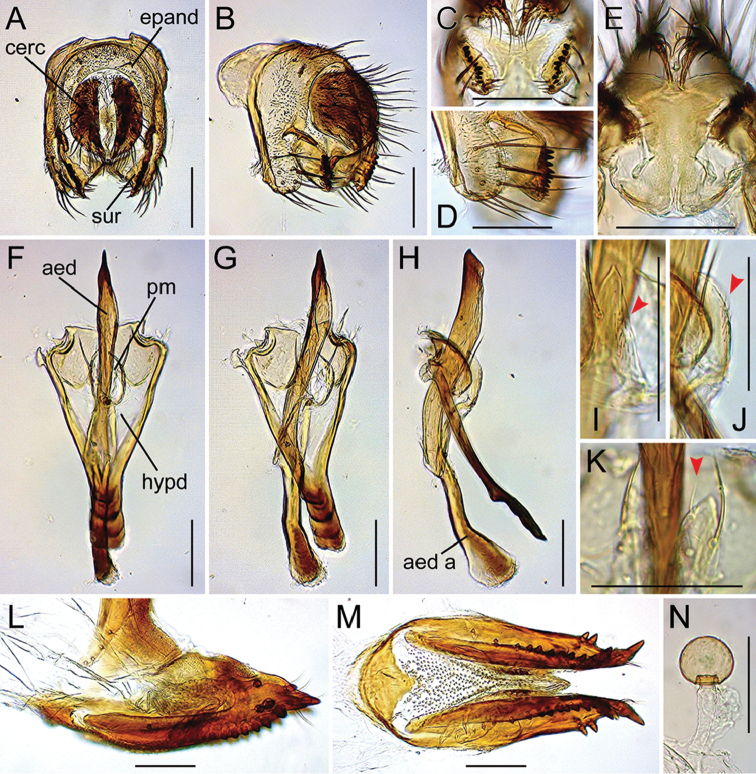
Drosophila (Dudaica) puberula sp. n. (**A–J** paratype #03366; **K** holotype #03365 **L–M** paratype #03426). **A, B** periphallic organs (caudal and caudolateral view, respectively) **C** surstyli (caudoventral view) **D** surstylus and epandrial ventral lobe (caudolateral view) **E** tenth sternite and ventral protrusions of cerci (ventral view) **F–H** phallic organs (ventral, ventrolateral, and lateral view, respectively) **I, J** pubescence (indicated with red arrowheads) and apical portion of parameres (ventrolateral and lateral view, respectively) **K** sensillum (indicated with red arrowhead) and apical portion of parameres (ventral view) **L, M** oviscapt (lateral and ventral view, respectively) **N** spermatheca. Scale bars: 0.1 mm.

**Figure 13.**
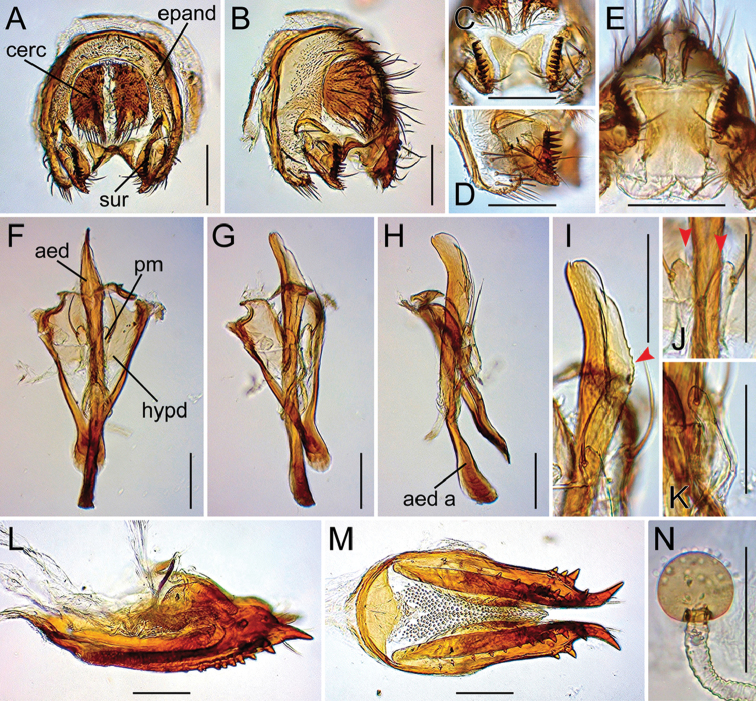
Drosophila (Dudaica) albipalpis sp. n. (**A–K** holotype #03908). **A, B** periphallic organs (caudal and caudolateral view, respectively) **C** surstyli (caudoventral view) **D** surstylus and epandrial ventral lobe (caudolateral view) **E** tenth sternite and ventral protrusions of cerci (ventral view) **F–H** phallic organs (ventral, ventrolateral, and lateral view, respectively) **I** apical portion of aedeagus with fine serrations (indicated with red arrowhead; ventrolateral view) **J, K** sensilla (indicated with red arrowheads) and apical portion of parameres (ventral and lateral view, respectively). Scale bars: 0.1 mm.

**Figure 14.**
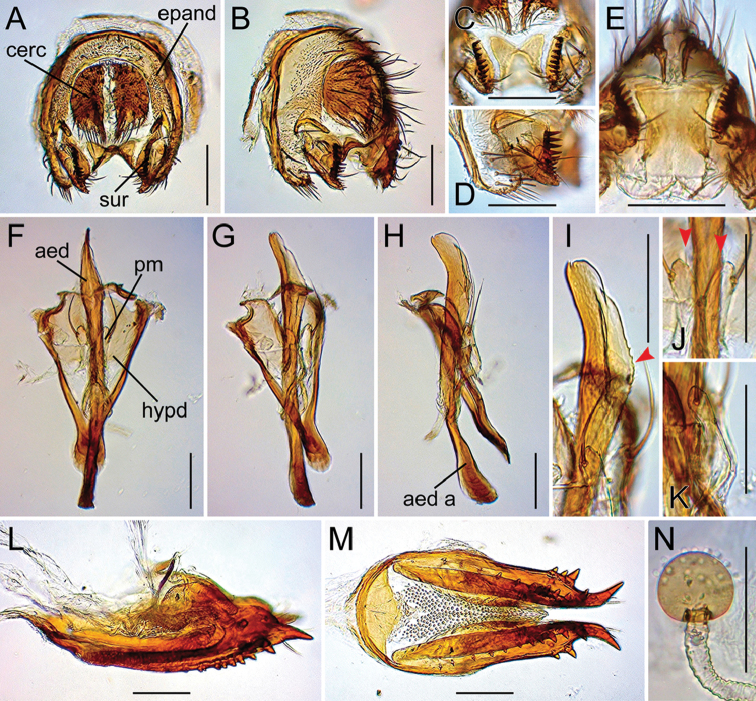
Drosophila (Dudaica) qiongzhouensis sp. n. (**A–K** paratype #03418 **L–N** paratype #03313). **A, B** periphallic organs (caudal and caudolateral view, respectively) **C** surstyli (caudoventral view) **D** surstylus and epandrial ventral lobe (caudolateral view) **E** tenth sternite and ventral protrusions of cerci (ventral view) **F–H** phallic organs (ventral, ventrolateral, and lateral view, respectively) **I** apical portion of aedeagus with fine serrations (indicated with red arrowhead; ventrolateral view) **J, K** sensilla (indicated with red arrowheads) and/or apical portion of parameres (ventral and lateral view, respectively) **L, M** oviscapt (lateral and ventral view, respectively) **N** spermatheca. Scale bars: 0.1 mm.

**Figure 15.**
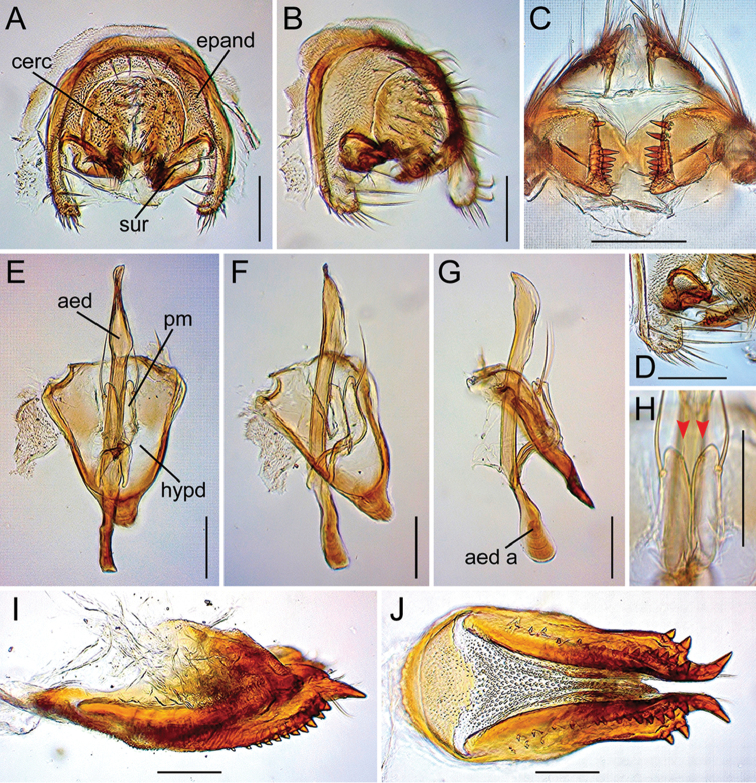
Drosophila (Dudaica) orthophallata sp. n. (**A–H** holotype #00177 **I, J** paratype #03906). **A, B** periphallic organs (caudal and caudolateral view, respectively) **C** surstyli, tenth sternite, and ventral protrusions of cerci (ventral view) **D** epandrial ventral lobe (caudolateral view) **E–G** phallic organs (ventral, ventrolateral, and lateral view, respectively) **H** sensilla (indicated with red arrowheads) and apical portion of parameres (ventral view) **I, J** oviscapt (lateral and ventral view, respectively). Scale bars: 0.1 mm.

**Figure 16.**
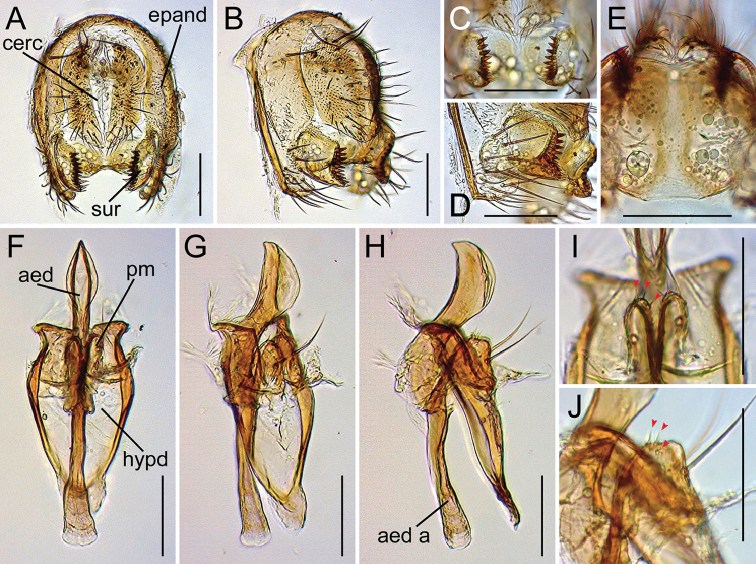
Drosophila (Dudaica) dissimilis sp. n. (**A–J** holotype #00430). **A, B** periphallic organs (caudal and caudolateral view, respectively) **C** surstyli (caudoventral view) **D** surstylus and epandrial ventral lobe (caudolateral view) **E** tenth sternite (ventral view) **F–H** phallic organs (ventral, ventrolateral, and lateral view, respectively) **I, J** sensilla (indicated with red arrowheads) and apical portion of parameres (ventral and lateral view, respectively). Scale bars: 0.1 mm.

*Male terminalia* (Figures 10B–K, 11B–L, 12A–K, 13, 14A–K, 15A–H, 16): Epandrium pale brown, pubescent except for anterior sub-dorsal to -ventral margin, ventrally narrowed, ventroapically rounded, with setae on caudodorsal and ventral portions; lobe-like apodeme present anterosubdorsally. Cercus dark brown, nearly entirely pubescent, separated from epandrium, caudoventrally with distinct process (except for *senilis* and *dissimilis* sp. n.). Surstylus more or less quadrangular; dorsoproximal portion broadly fused to epandrium, with sclerotized ridge connecting epandrium and surstylus (except for *dissimilis* sp. n.); outer surface not pubescent, anterosubmedially concaved; caudal margin with a slightly sinuate row of peg-like, apically more or less roundish prensisetae decreasing in size ventrally; ventral portion with apically pointed spines on either inner or outer surface: spines on inner, subventral surface longer, somewhat curved upwards. Tenth sternite pale brown, moderately sclerotized, anteromedially wrinkled (flat in *dissimilis* sp. n.). Hypandrium narrowly triangular (except for *orthophallata* sp. n. and *dissimilis* sp. n.), anteriorly with narrow, well developed apodeme, not pubescent (except for *malayana*, *gracilipalpis* sp. n., and *puberula* sp. n.), caudolaterally with a pair of somewhat expanded lobes; a pair of paramedian setae present on the portion fused to paramere. Paramere elongated, apically rounded in ventral view (except for *puberula* sp. n.), ventrosubapically with 1–2 sensilla (three in *dissimilis* sp. n.), basally fused to aedeagus, ventrally fused to hypandrium. Aedeagus rod-like, fused to aedeagal apodeme, pale brown to brown, apically darker, slightly curved dorsad (straight in *orthophallata* sp. n.); aedeagal guide and basal processes absent; apodeme anteriorly expanded in lateral view, shorter than aedeagus.

*Female terminalia* (Figures 10L–N, 11M–O, 12L–N, 14L–N, 15I, J): Tergite 8 pale brown, pubescent dorsally to caudolaterally. Epiproct dark brown, entirely pubescent. Hypoproct dark brown, laterally pubescent. Sternite 7 grayish brown, caudally darker, caudomedially deeply notched, nearly entirely pubescent; setae on caudal portion rather long. Oviscapt valve with stout lateral and marginal ovisensilla increasing in size posteriad; apical ovisensillum stout and the largest, bent outwards. Spermathecal capsule pale brown, spherical.

#### Included species.

*senilis* Duda, *malayana* (Takada), *gracilipalpis* Katoh & Gao, sp. n., *puberula* Katoh & Gao, sp. n., *albipalpis* Katoh, Toda & Gao, sp. n., *qiongzhouensis* Katoh & Gao, sp. n., *orthophallata* Katoh, Toda & Gao, sp. n., and *dissimilis* Katoh & Gao, sp. n.

##### Key to the species

In this key, not only morphological characters but also the selected “pure” diagnostic nucleotide sites of COI are used to distinguish between *albipalpis* sp. n. and *qiongzhouensis* sp. n. (see also Table 2). The numbers of cited figures of Duda (1926) and Gupta and Sundaran (1990) are given as figure D26 and figure G&S90, respectively.

**Table:** 

1	Palpus sinuate, with several stout setae only on basal portion (Figures 4A, B, 9A, B; figure D26–13)	**2**
–	Palpus nearly straight, with several stout setae scattered on entire length (Figures 11C–G, 16C–G)	**4**
2	Cercus without distinct caudoventral process (figure G&S90–2E)	***senilis* Duda, 1926**
–	Cercus with distinct caudoventral process (Figures 10F, 11F)	**3**
3	Foreleg tibia with two apical setae (Figure 10A)	***malayana* (Takada, 1976)**
–	Foreleg tibia with one apical seta (Figure 11A)	***gracilipalpis* Katoh & Gao, sp. n.**
4	Palpus medio- to baso-laterally dark grayish brown, with one apical, stout, prominent seta (Figure 9G); postpronotal lobe brownish white in upper half, dark brown in lower half (Figure 3C); notopleuron brownish white (Figure 3C); cercus without caudoventral process (Figure 16C, E)	***dissimilis* Katoh & Gao, sp. n.**
–	Palpus entirely white, without apical, prominent seta (Figure 9C–F); postpronotal lobe entirely dark brown (Figures 2C, D, 3A, B); notopleuron dark brown (Figures 2C, D, 3A, B); cercus with distinct caudoventral process (Figures 12–14E, 7C)	**5**
5	Palpus with 3–4 prominent, stout setae on outer, lateral surface (Figure 9C); hypandrium pubescent on the portion fused to paramere (Figure 12I, J); paramere apically sharp (Figure 12I–K); female abdominal tergite 8 caudodorsally lacking setae	***puberula* Katoh & Gao, sp. n.**
–	Palpus with many setae varied in size on outer, lateral surface (Figure 9D–F); hypandrium not pubescent; paramere apically round in ventral view, round or truncate in lateral view (Figures 13J, K, 14J, K, 15G, H); female abdominal tergite 8 caudodorsally with 3–4 setae	**6**
6	Aedeagus apically without small, acute claw, subapically swollen in lateral view (Figure 15G); oviscapt valve with 17–19 marginal ovisensilla (Figure 15I, J)	***orthophallata* Katoh, Toda & Gao, sp. n.**
–	Aedeagus apically with small, acute claw, subapically not swollen in lateral view (Figures 13H, I, 14H, I); oviscapt valve with ca. 14 marginal ovisensilla (Figure 14L, M)	**7**
7	Palpus broad, flat, not so rod-shaped in lateral view (Figure 9D); paramere apically truncated in lateral view (Figure 13K); nucleotide status in COI sequence = T (thymidine), T, C (cytosine), and T at sites 92, 226, 391, and 589, respectively (Table 3)	***albipalpis* Katoh, Toda & Gao, sp. n.**
–	Palpus slender, not so flat, somewhat rod-shaped in lateral view (Figure 9E); paramere apically rounded in lateral view (Figure 14K); nucleotide status in COI sequence = C, C, T, and C at sites 92, 226, 391, and 589, respectively (Table 3)	***qiongzhouensis* Katoh & Gao, sp. n.**

### Drosophila (Dudaica) malayana

Taxon classificationAnimaliaDipteraLauxaniidae

(Takada, 1976)

Figures 2A , 4 , 5 , 6A , 8A , 9A , 10

Zygothrica malayana Takada, 1976: 68.
Drosophila (Dudaica) malayana : Grimaldi 1990a: 30.

#### Specimens examined.

MALAYSIA: 2♂ (holotype and paratype of *Zygothricamalayana* Takada, 1976), near Kuala Lumpur, Peninsular Malaysia, 3.vii.1972, H Takada (SEHU); 2♂, Poring, Sabah, 16.iii.1999, MJ Toda (KPSP, SEHU); 1♂ (#03904), same except for 13.iii.2008 (KIZ); 1♂ (#03903), same except for 20.iii.2008 (SEHU). INDONESIA: 1♂, 1♀, Gunung Poteng, West Kalimantan, 4.xii.1996, MJ Toda (MZB, SEHU).

#### Diagnosis.

Palpus long, sinuate, with several stout setae only on basal portion (Figure 9A). Foreleg tibia with two apical setae (Figure 10A). Cercus with distinct caudoventral process (Figure 10F). Hypandrium pubescent on caudolateral lobes (Figure 10J).

#### Supplementary and revised description.

Adult ♂ and ♀. *Head* (Figures 2A, 4–6A, 8A, 9A): Arista with 7–8 dorsal and four ventral branches. Supracervical setae 25–30 per side; postocular setae 15–16 per side. Cibarium with ca. eight medial and ca. ten posterior sensilla per side. Prementum with five (one proximal, two lateral, and two distal) pairs of setae.

*Thorax* (Figure 2A): Postpronotal lobe milky white in upper half, dark brown in lower half, with 2–3 prominent but no short setae. Right and left dorsocentral setae nearly parallel. Notopleuron milky white. Thoracic pleura with three, sometimes rather indistinct stripes.

*Legs* (Figures 2A, 10A): Foreleg first tarsomere shorter than total length of four succeeding tarsomeres. Mid- and hind-leg first tarsomeres shorter than or as long as total length of four succeeding tarsomeres.

*Abdomen* (Figure 2A): Anterior bands on tergite 6 medially broadly interrupted.

*Male terminalia* (Figure 10B–K): Epandrium with ca. three and ca. 13 long setae per side on caudodorsal and ventral portions, respectively. Cercus with 23–24 setae. Surstylus with ca. nine prensisetae and 7–8 ventral spines. Paramere apically rounded in lateral view, with 1–2 sensilla. Aedeagus dorsoapically with a small, acute claw; apodeme slightly longer than 1/2 length of aedeagus.

*Female terminalia* (Figure 10L–N): Tergite 8 with two small setae on ventral portion but no on caudodorsal portion. Oviscapt valve yellowish brown, with three lateral, 13 marginal ovisensilla, and four (three dorsal, one ventral) subterminal, inner, trichoid ovisensilla. Spermathecal capsule slightly longer than broad; introvert ca. 1/5 height of capsule.

*Measurements* (in mm): BL (straight distance from anterior edge of pedicel to tip of abdomen) = 2.13–2.39/2.27 (range in 2♂/1♀ specimens), ThL (distance from anterior notal margin to apex of scutellum) = 0.98–1.08/1.01, WL (distance from humeral cross vein to wing apex) = 1.78–1.92/1.73, WW (maximum wing width) = 0.89–0.96/0.86.

*Indices*. FW/HW (frontal width/head width) = 0.56–0.60 (range in 2♂ and 1♀ specimens), ch/o (maximum width of gena/maximum diameter of eye) = 0.08–0.12, prorb (proclinate orbital seta/posterior reclinate orbital seta in length) = 0.61–0.62, rcorb (anterior reclinate orbital seta/posterior reclinate orbital seta in length) = 0.29–0.32, vb (subvibrissa/vibrissa in length) = 0.41–0.59, orbito (distance between proclinate and posterior reclinate orbital setae/distance between inner vertical and posterior reclinate orbital setae) = 0.50–0.67, dcl (anterior dorsocentral seta/posterior dorsocentral seta in length) = 0.64–0.71, sctl (basal scutellar seta/apical scutellar seta in length) = 0.95–0.98, sterno (anterior katepisternal seta/posterior katepisternal seta in length) = 0.59–0.74, dcp (distance between ipsilateral dorsocentral setae/distance between anterior dorsocentral setae) = 0.39–0.44, sctlp (distance between ipsilateral scutellar setae/distance between apical scutellar setae) = 0.94–1.09, C (2nd costal section between subcostal break and R_2+3_/3rd costal section between R_2+3_ and R_4+5_) = 1.75–1.81, 4c (3rd costal section between R_2+3_ and R_4+5_/M_1_ between r-m and dm-cu) = 1.25–1.36, 4v (M_1_ between dm-cu and wing margin/M_1_ between r-m and dm-cu) = 1.75–1.95, 5x (CuA_1_ between dm-cu and wing margin/dm-cu between M_1_ and CuA_1_) = 2.12–2.25, ac (3rd costal section between R_2+3_ and R_4+5_/distance between distal ends of R_4+5_ and M_1_) = 2.56–3.00, M (CuA_1_ between dm-cu and wing margin/M_1_ between r-m and dm-cu) = 0.75–0.86, C3F (length of heavy setation in 3rd costal section/length of 3rd costal section) = 0.50–0.62.

#### Distribution.

Malaysia (Peninsular Malaysia, Sabah*), Indonesia* (West Kalimantan). *New records.

#### Remarks.

*Drosophilamalayana* was originally described based only on male specimens collected from Peninsular Malaysia, with illustrations but only a very brief description of male terminalia (Takada 1976). We examined the type specimens of this species, and found that they share the following specific characters with the specimens collected from Borneo (Sabah and West Kalimantan): two strong setae present apically on foreleg tibia and distinctly pubescent caudolateral lobes of hypandrium. Based on these morphological characters, we identified the latter specimens as *D.malayana*, and described the female terminalia for the first time. However, our identification needs to be confirmed by DNA barcode data of additional specimens from the type locality in the future.

### Drosophila (Dudaica) gracilipalpis

Taxon classificationAnimaliaDipteraLauxaniidae

Katoh & Gao sp. n.

http://zoobank.org/A1FE2033-50A4-40D8-BAD0-0BA5B93B4890

Figures 2B , 4 , 5 , 6B , 8B , 9B , 11

#### Type material.

*Holotype* ♂ (#00484): CHINA: Wangtianshu, Mengla, Yunnan, ca. 670 m a.s.l., 22‒25.iv.2007, JJ Gao (KIZ).

*Paratypes.* INDONESIA: 1♂ (#03902), Bogor, West Java, 14‒15.xi.2009, MJ Toda (MZB). CHINA: 1♂ (#00033), Xishuangbanna Tropical Botanical Garden, Mengla, Yunnan, ca. 650 m a.s.l., 19.iii.2006, MJ Toda & KT Takano (KIZ); 1♂ (#03423), same except for 27‒28.ix.2011, JJ Gao (KIZ); 1♂ (#06001), same except for 28.ix.2011, JJ Gao (KIZ); 1♀ (#00485), same data as holotype (KIZ); 4♂, 1♀ (#00491, #00492, #03364, #03424, #03425), same except for 30.ix.2011 (KIZ).

#### Diagnosis.

Palpus long, sinuate, with several stout setae only on basal portion (Figure 9B). Foreleg tibia with one apical seta (Figure 11A). Cercus with distinct caudoventral process (Figure 11F). Hypandrium pubescent, but very indistinctly, on caudolateral lobes (Figure 11J).

#### Description

(characters in common with *D.malayana* not repeated). Adult ♂ and ♀. *Head* (Figures 2B, 4–6B, 8B, 9B): Arista with 7–8 dorsal and 3–4 ventral branches. Supracervical setae 26–27 per side; postocular setae 12–14 per side. Cibarium with ca. ten medial and ca. nine posterior sensilla per side. Prementum with six (one proximal, three lateral, and two distal) pairs of setae.

*Thorax* (Figure 2B): Postpronotal lobe with two prominent and rarely 1–2 short setae. Stripes on thoracic pleura mostly confluent with each other.

*Abdomen* (Figure 2B): Anterior bands on tergites 5 and/or 6 (and 7 in female) medially broadly interrupted.

*Male terminalia* (Figure 11B–L): Epandrium with ca. two and 10–12 long setae per side on caudodorsal and ventral portions, respectively. Cercus with 22–24 setae. Surstylus with 8–9 prensisetae and ca. seven ventral spines. Paramere ventrosubapically with one sensillum (longer in Indonesian specimen than in Chinese ones). Aedeagal apodeme ca. 1/2 length of aedeagus.

*Female terminalia* (Figure 11M–O): Tergite 8 with 4–5 small setae on ventral portion but no on caudodorsal portion. Oviscapt valve with three lateral and 12–13 marginal ovisensilla. Spermathecal capsule apically slightly narrowed, slightly broader than long.

*Measurements* (in mm): BL = 2.20 in holotype (range in 8♂/2♀ paratypes: 1.63–2.50/2.03–2.63), ThL = 1.00 (0.88–1.06/0.98–1.06), WL = 1.84 (1.60–2.15/2.03–2.10), WW = 0.92 (0.83–1.04/1.02–1.06).

*Indices*. FW/HW = 0.59 (range in 8♂ and 2♀, or less if noted, paratypes: 0.58–0.63), ch/o = 0.12 (0.07–0.13), prorb = 0.65 (6♂, 2♀: 0.46–0.63), rcorb = 0.30 (7♂, 2♀: 0.20–0.37), vb = 0.45 (7♂, 2♀: 0.22–0.60), orbito = 0.63 (0.50–0.64), dcl = 0.72 (0.63–0.79), sctl = 1.26 (7♂, 1♀: 0.94–1.12), sterno = 0.56 (0.44–0.71), dcp = 0.38 (0.41–0.57), sctlp = 0.95 (0.91–1.00), C = 1.70 (1.56–2.00), 4c = 1.45 (1.32–1.58), 4v = 2.05 (1.83–2.26), 5x = 2.38 (1.80–2.43), ac = 2.90 (2.87–3.29), M = 0.90 (0.75–0.95), C3F = 0.56 (0.52–0.59).

#### Distribution.

Indonesia (West Java), China (Yunnan).

#### Etymology.

Referring to the long and thin palpus.

#### Remarks.

This species closely resembles the foregoing species, *D.malayana*, in having the long, sinuate palpus and pubescent caudolateral lobe of hypandrium, but can be distinguished from the latter by having only one apical seta on foreleg tibia.

### Drosophila (Dudaica) puberula

Taxon classificationAnimaliaDipteraLauxaniidae

Katoh & Gao sp. n.

http://zoobank.org/94250D73-99A0-4C92-958C-5C1BFA018C08

Figures 2C , 4 , 5 , 6C , 8C , 9C , 12

#### Type material.

*Holotype* ♂ (#03365): CHINA: Xishuangbanna Tropical Botanical Garden, Mengla, Yunnan, ca. 650 m a.s.l., 27–28.ix.2011, JJ Gao (KIZ).

*Paratypes.* CHINA: 1♀ (#03426), same except for 19.iii.2006, MJ Toda and K Takenaka (KIZ); 5♂, Wangtianshu, Mengla, Yunnan, ca. 670 m a.s.l., 22–25.iv.2007 (#00480) or 30.ix.2011 (#03366–69), JJ Gao (KIZ, SEHU).

#### Diagnosis.

Palpus slightly shorter than arista, with 3–4 prominent, stout setae on outer lateral surface (Figure 9C). Hypandrium pubescent on portion fused to paramere (Figure 12I, J). Paramere apically sharp (Figure 12I–K).

#### Description

(characters in common with *D.gracilipalpis* sp. n. not repeated). Adult ♂ and ♀. *Head* (Figures 2C, 4–6C, 8C, 9C): Arista with 6–8 dorsal and 2–3 ventral branches. Supracervical setae 20–21 per side; postocular setae 15–18 per side. Cibarium with ca. nine medial and ca. eight posterior sensilla per side. Prementum with 5–6 (one proximal, 2–3 lateral, and two distal) pairs of setae.

*Thorax* (Figure 2C): Postpronotal lobe entirely dark brown, with 1–3 prominent and 2–4 short setae. Right and left dorsocentral setae slightly convergent. Notopleuron dark brown. Thoracic pleura with four stripes.

*Legs* (Figure 2C): First tarsomeres of all legs shorter than total length of four succeeding tarsomeres; mid-leg first tarsomere with one subproximal short, black spine.

*Male terminalia* (Figure 12A–K): Epandrium with ca. three and 10–13 long setae per side on caudodorsal and ventral portions, respectively. Cercus with 33–35 setae. Surstylus with ca. nine prensisetae and ca. eight ventral spines. Hypandrium not pubescent on caudolateral lobes. Paramere apically with one sensillum. Aedeagus apically darkened; apodeme laterally flat in muscle-attaching portion.

*Female terminalia* (Figure 12L–N): Tergite 8 with ca. four small setae on ventral portion but no on caudodorsal portion. Oviscapt valve with three lateral and 13–14 marginal ovisensilla. Spermathecal capsule apically round, not narrowed.

*Measurements* (in mm): BL = 2.30 in holotype (range in 5♂/1♀ paratypes: 1.92–2.43/2.69), ThL = 0.88 (0.98–1.14/1.30), WL = 1.86 (1.82–2.10/2.35), WW = 0.98 (0.88–1.10/1.20).

*Indices.* FW/HW = 0.57 (range in 5♂ and 1♀, or less if noted, paratypes: 0.55–0.58), ch/o = 0.11 (0.07–0.18), prorb = 0.61 (0.54–0.58), rcorb = 0.30 (5♂: 0.27–0.38), vb = 0.34 (0.33–0.41), orbito = 0.63 (0.50–0.75), dcl = 0.65 (4♂, 1♀: 0.55–0.74), sctl = 1.05 (4♂, 1♀: 1.00–1.10), sterno = 0.67 (5♂: 0.42–0.67), dcp = 0.52 (0.42–0.55), sctlp = 1.05 (0.78–1.00), C = 1.67 (1.50–1.79), 4c = 1.43 (1.36–1.58), 4v = 2.00 (1.75–2.16), 5x = 1.70 (1.79–2.11), ac = 3.00 (2.83–3.58), M = 0.81 (0.76–0.89), C3F = 0.55 (0.53–0.59).

#### Distribution.

China (Yunnan).

#### Etymology.

Referring to the pubescence of hypandrium on the portion fused to paramere in the new species.

### Drosophila (Dudaica) albipalpis

Taxon classificationAnimaliaDipteraLauxaniidae

Katoh, Toda & Gao sp. n.

http://zoobank.org/BC85BE2C-0720-4B19-BCF5-2BB361F96BC5

Figures 2D , 4 , 5 , 6D , 8D , 9D , 13

#### Type material.

*Holotype* ♂ (#03908): INDONESIA: Cikaniki, Gunung Halimun, West Java, 4.xi.2009, MJ Toda (MZB).

#### Diagnosis.

Palpus broad, flat, with a few moderate setae on outer lateral surface (Figure 9D). Paramere apically truncated in lateral view (Figure 13K). Aedeagus apically finely serrated along ventrolateral margin (Figure 13I). Nucleotide status in COI sequence = C and T at sites 391 and 589, respectively (Table 3).

#### Description

(characters in common with *D.puberula* sp. n. not repeated). Adult ♂. *Head* (Figures 2D, 4–6D, 8D, 9D): Arista with eight dorsal and three ventral branches. Supracervical setae 24–27 per side; postocular setae 14–17 per side. Cibarium with ca. nine medial and ca. 13 posterior sensilla per side. Prementum with five (one proximal, two lateral, and two distal) pairs of setae.

*Thorax* (Figure 2D): Postpronotal lobe with two prominent and three short setae. Anterior dorsocentral setae slightly convergent; posterior dorso-central setae nearly parallel. Thoracic pleura with four indistinct stripes.

*Legs* (Figures 2D): First tarsomeres of all legs slightly shorter than total length of four succeeding tarsomeres.

*Male terminalia* (Figure 13): Cercus with 29–32 setae, including ca. ten ventral, small ones. Surstylus with ca. ten prensisetae and ca. eleven ventral spines. Hypandrium not pubescent. Paramere apically rounded in ventral view, with 1–2 sensilla. Aedeagus apically not darkened.

*Measurements* (in mm): BL = 2.50 in holotype, ThL = 1.08, WL = 2.10, WW = n/a.

*Indices.* FW/HW = 0.59, ch/o = 0.09, prorb = 0.63, rcorb = 0.24, vb = n/a, orbito = 0.50, dcl = 0.67, sctl = 1.02, sterno = 0.70, dcp = 0.50, sctlp = 0.91, C = 1.71, 4c = 1.31, 4v = 1.65, 5x = 1.38, ac = 2.83, M = 0.69, C3F = 0.49.

#### Distribution.

Indonesia (West Java).

#### Etymology.

Referring to the white palpus in the new species.

### Drosophila (Dudaica) qiongzhouensis

Taxon classificationAnimaliaDipteraLauxaniidae

Katoh & Gao sp. n.

http://zoobank.org/EBC67347-3CC4-4598-A9AC-248A04D7571F

Figures 3A , 4E , 5E , 7A , 8E , 9E , 14

#### Type material.

*Holotype* ♂ (#03420): CHINA: Jianfengling National Nature Reserve, Ledong, Hainan, ca. 750 m a.s.l., 17–20.iv.2008, JJ Gao (KIZ).

*Paratypes*. CHINA: 5♂, 4♀ (#03310–15, #03418, #03419, #03422), same data as holotype (KIZ, SEHU).

#### Diagnosis.

Palpus slender, not so flat, somewhat rod-shaped in lateral view, with a few moderate setae on outer lateral surface (Figure 9E). Paramere apically rounded in lateral view (Figure 14K). Nucleotide status in COI sequence = C and C at sites 92 and 226, respectively (Table 3).

#### Description

(characters in common with *D.puberula* sp. n. not repeated). Adult ♂ and ♀. *Head* (Figures 3A, 4E, 5E, 7A, 8E, 9E): Arista with 6–8 dorsal and three ventral branches. Supracervical setae 23–24 per side; postocular setae 15–17 per side. Cibarium with ca. ten medial and ca. nine posterior sensilla per side. Prementum with five (one proximal, two lateral, and two distal) pairs of setae.

*Thorax* (Figure 3A): Postpronotal lobe with 1–2 prominent and 1–3 short setae.

*Legs* (Figure 3A): Foreleg first tarsomere shorter than total length of four succeeding tarsomeres. Mid- and hind-leg first tarsomeres slightly shorter than or nearly as long as total length of four succeeding tarsomeres.

*Male terminalia* (Figure 14A–K): Epandrium caudodorsally with 2–3 and ca. 16 long setae per side on caudodorsal and ventral portions, respectively. Cercus with 31–33 setae. Surstylus with ca. nine prensisetae and ca. ten ventral spines. Hypandrium not pubescent. Paramere apically rounded in ventral view, with one sensillum. Aedeagus apically not darkened, subapically sometimes scarcely serrated along ventrolateral margin.

*Female terminalia* (Figure 14L–N): Tergite 8 with 4–5 and ca. four setae on ventral and caudodorsal portions, respectively. Oviscapt valve with 3–4 lateral and ca. 14 marginal ovisensilla. Spermathecal introvert ca. 1/6 height of capsule.

*Measurements* (in mm): BL = 2.72 in holotype (range in 5♂/4♀ paratypes: 1.99–2.36/2.08–3.00), ThL = 1.22 (0.90–0.98/0.82–1.19), WL = 2.30 (1.74–1.82/1.62–2.33), WW = 1.26 (0.88–0.96/0.82–1.24).

*Indices.* FW/HW = 0.58 (range in 5♂ and 4♀, or less if noted, paratypes: 0.55–0.58), ch/o = 0.09 (0.08–0.13), prorb = 0.68 (5♂, 3♀: 0.50–0.80), rcorb = 0.36 (0.27–0.45), vb = 0.40 (0.32–0.55), orbito = 0.70 (0.50–0.67), dcl = 0.67 (3♂, 4♀: 0.58–0.68), sctl = 0.96 (0.89–1.06), sterno = 0.47 (0.54–0.68), dcp = 0.49 (0.42–0.52), sctlp = 0.83 (0.69–1.00), C = 1.66 (1.48–1.87), 4c = 1.46 (1.20–1.61), 4v = 1.88 (1.62–2.00), 5x = 1.36 (1.45–1.90), ac = 2.92 (2.90–3.44), M = 0.73 (0.68–0.83), C3F = 0.61 (0.51–0.62).

#### Distribution.

China (Hainan).

#### Etymology.

Pertaining to the type locality, Hainan (formerly known as “Qiongzhou”).

### Drosophila (Dudaica) orthophallata

Taxon classificationAnimaliaDipteraLauxaniidae

Katoh, Toda & Gao sp. n.

http://zoobank.org/BF65BAAF-9A66-4ECD-B4CE-65723DDAB9AE

Figures 3B , 4F , 5F , 7B , 8F , 9F , 15

#### Type material.

*Holotype* ♂ (#00177): MALAYSIA: Ulu Senagang, Crocker Range, Sabah, 18.x.1999, MJ Toda (ITBC).

*Paratypes*. MALAYSIA: 2♀ (#03905, #03906), Park Headquarters, Mt. Kinabalu, Sabah, 11.iii.2008, MJ Toda (KPSP, SEHU).

#### Diagnosis.

Palpus broad, flat, with a few moderate setae on outer lateral surface (Figure 9F). Paramere apically somewhat truncated in lateral view (Figure 15G). Aedeagus straight, subapically swollen in lateral view, apically without small, acute claw (Figure 15G).

#### Description

(characters in common with *D.puberula* sp. n. not repeated). Adult ♂ and ♀. *Head* Figures 3B, 4F, 5F, 7B, 8F, 9F): Arista with 6–7 dorsal and 2–3 ventral branches. Supracervical setae 25–26 per side; postocular setae 16–18 per side. Cibarium with ca. nine medial and ca. ten posterior sensilla per side. Prementum with five (one proximal, two lateral, and two distal) pairs of setae.

*Thorax* (Figure 3B): Postpronotal lobe with 1–2 prominent and 1–3 short setae. Thoracic pleura with four, slightly indistinct stripes.

*Legs* (Figure 3B): Foreleg first tarsomere shorter than or as long as total length of four succeeding tarsomeres. Mid- and hind-leg first tarsomeres as long as total length of four succeeding tarsomeres.

*Male terminalia* (Figure 15A–H): Epandrium with ca. three and ca. 13 long setae per side on caudodorsal and ventral portions, respectively. Cercus with 26–28 setae. Surstylus with ca. eight prensisetae and 7–8 ventral spines. Tenth sternite damaged. Hypandrium somewhat triangular, anteriorly round, not pubescent. Aedeagus apically not darkened; apodeme ca. 2/5 length of aedeagus.

*Female terminalia* (Figure 15I, J): Tergite 8 with 3–4 and ca. three setae on ventral and caudodorsal portions, respectively. Oviscapt valve with three lateral and 17–19 marginal ovisensilla. Data of spermatheca unavailable.

*Measurements* (in mm): BL = 2.34 in holotype (range in 2♀ paratypes: 2.20–2.60), ThL = 1.18 (1.18–1.40), WL = 2.00 (2.35–2.63), WW = 1.06 (1.28–1.37).

*Indices.* FW/HW = 0.56 (range in 2♀, or less if noted, paratypes: 0.56–0.63), ch/o = 0.09 (0.12), prorb = 0.68 (1♀: 0.67), rcorb = 0.34 (1♀: 0.31), vb = n/a (1♀: 0.50), orbito = 0.65 (0.40–0.55), dcl = 0.65 (0.71–0.75), sctl = 1.00 (1♀: 1.04), sterno = 0.58 (0.63–0.71), dcp = 0.44 (0.43), sctlp = 0.72 (0.73–0.85), C = 1.72 (1.76–1.78), 4c = 1.25 (1.27–1.28), 4v = 1.61 (1.59–1.67), 5x = 1.33 (1.33–1.44), ac = 3.56 (2.93–3.04), M = 0.63 (0.67–0.72), C3F = 0.56 (0.54–0.58).

#### Distribution.

Malaysia (Sabah).

#### Etymology.

Referring to the straight aedeagus in the new species.

#### Remarks.

The paratype female specimens #03905 and #03906 were identified as conspecific with the holotype male specimen #00177 (DNA sequence data unavailable), based on close morphological affinity between them. This species can also be distinguished from the other *Dudaica* species by oviscapt valve with 17–19 marginal ovisensilla (Figure 15I, J) in addition to the diagnosis.

### Drosophila (Dudaica) dissimilis

Taxon classificationAnimaliaDipteraLauxaniidae

Katoh & Gao sp. n.

http://zoobank.org/BABEAEB4-04FD-43CF-B3BC-60B69F3FE280

Figures 3C , 4G , 5G , 7C , 8G , 9G , 16

#### Type material.

*Holotype* ♂ (#00430): CHINA: Hesong, Xiding, Menghai, Yunnan, ca. 1,900 m a.s.l., 7.iv.2011, JJ Gao (KIZ).

#### Diagnosis.

Palpus short, club-shaped, medio- to baso-laterally dark grayish brown, with one prominent seta apically and several long setae ventrally (Figure 9G). Cercus without caudoventral process (Figure 16E). Paramere apically somewhat quadrate in lateral view (Figure 16J), ventroapically with three sensilla (Figure 16I, J). Aedeagus distally dilated laterally, somewhat lunate in lateral view (Figure 16F–H).

#### Description

(characters in common with *D.orthophallata* sp. n. not repeated). Adult ♂. *Head* (Figures 3C, 4G, 5G, 7C, 8G, 9G): Longest axis of eye nearly rectangular to body axis. Frontal vitta grayish white. Fronto-orbital plate slightly grayish; anterior reclinate orbital seta situated between proclinate and posterior reclinate orbital setae. Occiput and postgena dark brown, marginally milky white. Arista with six dorsal and three ventral branches. Supracervical setae 16–19 per side; postocular setae 17–19 per side. Cibarium slightly thickened on anterior margin; medial sensilla ca. nine per side and posterior sensilla ca. nine per side; first and second medial sensilla weaker than and anteriorly apart from others. Clypeus not thickened at median portion, laterally dark brown.

*Thorax* (Figure 3C): Postpronotal lobe pale brownish white in upper half, dark brown in lower half; setae broken. Dorsocentral and scutellar setae broken. Notopleuron pale brownish white. Thoracic pleura nearly entirely dark brown, without stripes.

*Wing* (Figure 3C) slightly wrinkled at basal portion of R_4+5_; longitudinal veins pale brown except for basal section of M_1_ (brown) and CuA_1_ (brown).

*Legs* (Figure 3C) pale grayish yellow to pale yellow. Foreleg first tarsomere shorter than total length of four succeeding tarsomeres. Mid- and hind-leg first tarsomeres slightly shorter than total length of four succeeding tarsomeres, without subproximal spine.

*Abdomen* (Figure 3C): Tergites pale brown, each with dark brown caudal band narrower than ca. 1/2 of tergite but laterally extended anteriorly.

*Male terminalia* (Figure 16): Epandrium with ca. two and ca. 16 long setae per side on caudodorsal and ventral portions, respectively. Cercus with 30–31 setae. Surstylus with ca. eight prensisetae and ca. ten ventral spines; basal sclerotized ridge indistinct. Tenth sternite flat, not wrinkled. Hypandrium somewhat oval in anterior portion. Aedeagal apodeme apically not flattened, slightly shorter than aedeagus.

*Measurements* (in mm): BL = 2.33 in holotype, ThL = 1.04, WL = 2.35, WW = 1.16.

*Indices.* FW/HW = 0.60, ch/o = 0.08, prorb = n/a, rcorb = n/a, vb = n/a, orbito = 0.78, dcl = n/a, sctl = n/a, sterno = 0.62, dcp = 0.65, sctlp = 0.94, C = 2.09, 4c = 1.21, 4v = 1.95, 5x = 1.24, ac = 2.83, M = 0.64, C3F = 0.53.

#### Distribution.

China (Yunnan).

#### Etymology.

Referring to the morphological difference from the other species in the subgenus Dudaica.

#### Remarks.

This species is the most different in morphology from the other *Dudaica* species, such as the shape of palpus and parameres, apical prominent seta on palpus, and number of sensilla on parameres. Those characters are also seen in many other species than *Dudaica*, suggesting the plesiomorphic states of these characters.

## Supplementary Material

XML Treatment for Subgenus Dudaica

XML Treatment for Drosophila (Dudaica) malayana

XML Treatment for Drosophila (Dudaica) gracilipalpis

XML Treatment for Drosophila (Dudaica) puberula

XML Treatment for Drosophila (Dudaica) albipalpis

XML Treatment for Drosophila (Dudaica) qiongzhouensis

XML Treatment for Drosophila (Dudaica) orthophallata

XML Treatment for Drosophila (Dudaica) dissimilis
